# Transitions in Black and Latinx Community-Based Doula Work in the US During COVID-19

**DOI:** 10.3389/fsoc.2021.611350

**Published:** 2021-03-11

**Authors:** Mariel Rivera

**Affiliations:** Department of Anthropology, Syracuse University, Syracuse, NY, United States

**Keywords:** COVID-19, Doulas, community-based doulas, Black women, Latinx women, New York State

## Abstract

In response to COVID-19, many doulas, including community-based doulas (CBDs), have shifted to virtual doula work, placing aspects of doula care online. CBDs typically center Black and Brown mothers and come from the same community as their clients, granting access to doula care for many individuals who would traditionally not have access. Two partner CBD organizations in Central New York—Village Birth International and Doula 4 a Queen—transitioned to virtual doula work, continuing to center Black and Afro-Latinx people. As CBDs began to transition their work online, they had to create new ways to include both the community and doula aspects of their work. My research has captured these doulas’ experiences since mid-2019 and has documented their transition from in-person doula work to virtual work. This also included their experiences of hosting doula trainings that were originally designed to be held in person. To understand this turn to virtual doula work, in this article I draw on social media engagement, online interviews, Zoom discussions, and personal experience to capture how CBD work shifted to virtual platforms can still center Black and Afro-Latinx folks in their communities and beyond.

## Introduction

COVID-19 has wholly changed the daily lives of billions of individuals worldwide. The virus carries uncertainties with it: How long will this last? Who will become infected? Will they live, or die? New terms like “social distancing” and “quarantine” have become a part of our daily lexicon as humanity figured out how to address this virus.

Updates and information about the virus and how to protect yourself from it seem to change daily. In the United States, the Center for Disease Control and Prevention (CDC) guidelines and State health departments compensated for the changes demanded by the virus by implementing new safety recommendations in all walks of life. At the beginning of the epidemic, most recommendations were to not gather in large groups, regularly wash your hands, and don't touch your face. These recommendations quickly changed to no gatherings outside of your household and only leaving home for essential items or trips. Then face masks became a necessity for engaging in essential functions outside the home. Birth, doula work, and community engagement have not been immune to the sorts of drastic changes that have come with the virus in the United States and globally.

New York State (NYS) had a rapid onset of COVID-19 cases that threatened to overwhelm the hospital system. Governor Cuomo and the State Health Department took dire measures in reaction to the virus to reduce the spread. There were significant changes in hospital-based maternity care, explicitly limiting the presence of in-person support, which directly impacted doula care. When the new maternal health policies emerged, I was in the midst of my dissertation research in Syracuse, New York, on Black and Brown community-based doulas (CBDs). Through this research, I gained an understanding of the quick adaptations doulas needed to make during a crisis to maintain their work and to express their views on the emerging policies of the local and state-level stakeholders.

Two partner CBD organizations I work with in Syracuse, New York—Village Birth International (VBI), and Doula 4 a Queen (D4Q)—transitioned to virtual doula work, continuing to center Black and Afro-Latinx folks and communities in their practice. As CBDs began to transition their work online, they had to create new ways to include both the community and individualized doula care aspects of their work. My research has captured these doulas’ experiences since mid-2019 and has documented their transition from in-person doula work to virtual work. This research included their experience of hosting a virtual doula training, originally designed to be in-person, and activist-based actions related to Black Lives Matter (BLM). The doulas began to implement virtual options for care and hosted “Live” meetings on their platforms. They also extended their social media campaigns and continued their activist engagement.

This research captures how CBD work shifted to virtual work and can still center Black and Afro-Latinx folks in their communities and beyond. To understand this adaptation towards online doula care, I draw on Zoom discussions, online interviews, social media engagements, and on my participant-observation experiences as a doula and activist member of both organizations. From my research, it is evident that guaranteeing that doula care remains accessible to all community members is fundamental to CBDs for moving forward throughout this extraordinary period. However, the doulas also needed to contemplate hospital protocols, state health policies, and their overall safety and that of their clients. Thus these doulas had to negotiate their work within a myriad of elements amidst a global health pandemic.

## The Black Doulas of Syracuse

Doulas are non-medical support people and have historically been seen at births around the world, usually as family members or knowledgeable community members (Campbell-Voytal et al., 2011). Contemporaneously, a doula is defined as a person who delivers non-medical support to an expectant mother before, during, and after birth. The support provided can include assisting the pregnant person to create a birth plan, breastfeeding counseling, perinatal information, and constant labor encouragement (Gordon et al., 1999; Chor et al., 2016). Continuous labor support has numerous benefits physically and emotionally for birthing people (Hodnett et al., 2013; Steel et al., 2015; Bohren et al., 2017). Doula-supported birth has been proven to reduce cesarean delivery (Chor et al., 2016; Toonen, 2018); improve breastfeeding (McLeish and Redshaw, 2018); decrease the use of pain medication (Gordon et al., 1999; Jordan et al., 2008); decrease labor time (Scott et al., 2000), and increase women’s overall satisfaction with their birth experiences (Thomas et al., 2017). For Black and other marginalized peoples, doulas can also help to overcome maternal health inequities and barriers while supporting a woman in having a safe and positive birth (Gruber et al., 2013; Haderman and Kozimannil, 2016). Generating more knowledge about doulas, especially doulas of color who work with Black and other marginalized peoples, is essential to understand the practice of doulas and to filling the literature gaps (Bohren et al., 2017).

Many Community-Based Doulas (CBDs) utilize a Reproductive Justice (RJ) framework within their practice, created by Black women and other women of color designed to center their lived reproductive experiences (Ross, 2017). The framework, created by Black women in the United States to talk about their reproductive experiences, goes beyond reproductive rights. The framework centers the right not to have a child, the right to have a child, and the right to parent that child, as well as sexual autonomy and gender freedom (Ross et al., 2017). In this way, RJ broadened the scope of reproductive rights. The creators of this framework wrote that RJ “created a radical shift from ‘choice’ to ‘justice’ to locate women’s autonomy as a self-determination in international human rights standards and laws rather than in the constitutionally limited concepts of individuals rights and privacy” (Ross et al., 2017:18). Consequently, RJ looks past the legal paradigm of reproductive rights to eliminate all reproductive freedom barriers, from social to economic. Many CBDs are Black women or other women of color, often live in the same community as their clients, and position their work to target maternal health disparities (Ross and Solinger, 2017).

Black women in the United States confront discrimination, both implied and overt, often leading them into a birth environment in which they are vulnerable and which contributes to adverse maternal health outcomes (Adams and Thomas, 2017). Currently, Black women are three to four times more likely to die perinatally than white women and face higher instances of maternal morbidity and prematurity (Center for Disease Control and Prevention (CDC), 2020). Generally, to combat these negative maternal health outcomes, CBDs separate their work from other kinds of doulas in particular ways. CBD work includes "all of the services that private doulas offer, and adds additional home visits and a wider array of services…CBDs have additional training that supplements the traditional doula education curriculum. Care provided is low or no cost and is grounded in safe, dignified and respectful access to health care" (Bey et al., 2019:9). Therefore, CBDs’ work maintains one-on-one support during pregnancy, birth, and the postpartum period and participates in broader community-oriented public health programming.

I work with two interrelated organizations in Syracuse—VBI, run by women of color, predominately Black women, and D4Q, a related CBD organization founded by a Black woman. These two CBD organizations employ an RJ framework and connect doulas to women who face social, economic, and medical oppression, tailoring their efforts towards Black women (Village Birth International (VBI), 2019a). Sequoia, the founder and a doula with D4Q, explained that "doula work means Black liberation…birth work [for us] isn't just about the nine months, or the six weeks postpartum. It's about the longevity of Black life." In this way, birth work for these doulas bring together prenatal and labor support, advocacy, and activism, demonstrating the RJ framework that is apparent in their doula practice. From my positionality as a woman of Puerto Rican and Dominican descent, I felt compelled to engage with work geared towards ensuring equity in maternal health care.

The CBDs value making doula services accessible for all, regardless of financial compensation. They have specific outreach for individuals who cannot afford doula services, such as offering sliding payment scales or “scholarships” that cover the entire cost. Parity varies: some birthing mothers have previous births, and for some, this is their first. These CBD organizations offer expectant women four prenatal meetings, birth plan counseling, uninterrupted labor support, and two postpartum visits without charge. In general, doulas practice in hospitals, birthing centers, and at home births. VBI and D4Q doulas attend births only in hospitals due to the absence of homebirth midwives in the Syracuse region. These organizations are affiliated with 10 community doulas, including myself, and are in the training process with others.

Through these organizations, women from the Syracuse community can learn about critical resources in the community, identify a doula for their own birth, and go through doula certification if they wish. Overall, CBD practice centers Black mothers and families in one-on-one care and within broader community efforts.

Community-based public health outreach is an integral part of both organizations' work. Thus, VBI and D4Q are committed to creating programming within and for the city of Syracuse. Syracuse demonstrates similar demographics as the rest of the country; it is an urban, mid-sized city with five hospitals (Onondaga County, 2016). Historically, vast inequalities have persisted in the Black community of Syracuse. The historical legacy of this segregation marks Syracuse with patterns of low birthweight (Lane et al., 2008); gun violence (Larsen et al., 2017); disproportionate rates of incarceration for Black men (Keefe et al., 2017); and food deserts (Lane et al., 2008). Asteir, a founding doula of VBI, emphasized that the health "disparities are based in a history of oppression and racism." Due to these continued health disparities in the Black community, the CBDs place significant emphasis on broad public health work. This broader community outreach includes programs such as the Black Healing Expo, which brought Black health experts together with the community. For these doulas, to have healthy Black mothers and families, doula work needs to operate not only during pregnancy, labor, or postpartum but also throughout the broader community.

In doing this work, both VBI and D4Q have remained autonomous, relying on grant funding with some individual payments from clients and community fundraising. They do not get reimbursed by Medicaid, and were critical of the proposed NYS beyond the Medicaid-reimbursement bill and certificate policies put forth last year (New York State (NYS), 2019). The Medicaid reimbursement bill was planned to allow enrollees of Medicaid fee-for-service and Medicaid Managed Care to have their doulas paid directly through the state and included standard doula practices for each doula to follow (New York State (NYS), 2019). Moreover, Assembly Bill A364B was introduced into the New York State Senate that looked to positively sanction specific doulas based on their state-regulated certification. The bill explicitly outlined the perimeters for certified doulas, including a fee, and only permitted certified doulas to provide doula services (New York State Senate (NYSS), 2019). Governor Cuomo ultimately vetoed the bill.

The CBDs had specific objections to how doulas became recognized by the state and to what they viewed as constraints to their practice. Specifically, these constraints included licensing fees, unknown curriculum for certification, and general state regulation of doulas. VBI published an open-letter critique of the bill, which stated, "The regulation and restriction of all doulas in NY State, and implementation of certification policies without incorporating community-based doula models, erase not only this legacy but the potential to save lives and support families with the dignity and culturally sensitive reproductive care they deserve" (Village Birth International (VBI), 2019b). It is evident that the regulation and certification policies put forth by state representatives are troublesome to existing CBDs. The history of the CBDs opposing NYS doula regulation is vital to understanding how VBI and D4Q reacted to NYS health policies in response to COVID-19.

The coronavirus left much CBD work in limbo because of the necessary restrictions on face-to-face interactions. CBDs developed new concerns about Black women's health, state regulation, and hospital policy in response to the virus's spread. Thus, just like the rest of the world, VBI and D4Q had to form immediate responses to COVID-19, starting with their bi-annual training held during mid-March of 2020.

## Methodology

I have worked with VBI and D4Q since 2018. During this time, I have attended and assisted in teaching childbirth education classes, observed client meetings with doulas, witnessed doulas' support of laboring parents, attended births and participated in birth activism. Between March 13th and May 15th, I collected data through interviews, virtual participant-observation, and social media cataloging. The interviews were semi-structured, open-ended, and guided by three main questions: How has COVID-19 influenced doula work? How have COVID-19 hospital/public health policies impacted doula work? What do doulas need to work in person safely? Because of the rapid nature of the interviews and the limitation of COVID-19, I only interviewed six doulas for approximately 1 h each. Informed consent was previously obtained from all doulas as well as permission from the organizations to collect data during events. The virtual participant-observations took place on Zoom or FaceTime during doula planning meetings, childbirth education courses, and doula trainings. I virtually attended four trainings, three meetings, and three childbirth education classes. The social media cataloging took place on Facebook and Instagram and included engaging with ‘Live’ on the platforms. I obtained ethical approval from Syracuse University, IRB #19-231.

All qualitative data including fieldnotes, interviews and social media posts from mid-March until August was coded for thematic elements. Through coding, the themes of the doulas' immediate responses to COVID-19, public health guidelines, clients, community needs, and the future of in-person services emerged. My discussion of the findings below begins by discussing the immediate responses to COVID-19, then considers what the doulas themselves highlight as a need for re-starting in-person services. This research looks to add to the small but growing body of literature on doulas.

## Immediate Response to the Virus

The onset of the coronavirus coincided with my dissertation research on these two Community-Based Doula (CBD) organizations. The beginning of March 2020 was a busy time for both organizations as there was a training planned for new doulas. During planning for the training, Asteir stressed that an essential part of becoming a doula is passing on your knowledge and creating a doula community. Every training is slightly different as new experiences occur, further information is released, and new techniques are shared. I assisted in organizing specific material for each weekend of the training, such as the Black Mamas Matter toolkit (Black Mamas Matter (BMMA), 2018), which consisted of a human rights-based approach to reproductive health for and by Black women. The training materials included discussion topics, events, films, and guest speakers. The majority of women who attended the training identified as Black, are mothers, and live in the Syracuse community. These women usually have ties to existing doulas trained by and working with VBI or D4Q doulas; some have been doula clients. Thus, many trainees come in with specific connections to doula work and reasons why they desire to become a doula in Syracuse working with Black women.

The doula training began on the first weekend the virus took hold in NYS—Friday, March 13th to Sunday, March 15th. Twelve trainees, two educators, and I spent the weekend in the community center in the Southside of Syracuse. Throughout the first cold and dark March evening, a “Welcome Dinner” took place to encourage participants to become familiar with each other. Talk about the virus sprinkled in with the usual small talk and introductions. Asteir, the lead trainer, discussed the decision to continue or postpone with the group. Most seemed adamant that they were not worried about the virus in such an intimate group and wanted to continue the training. Some cited that they could not take off more time for work if the days changed, as most had full-time jobs. At this point, we did not fully understand how the virus would alter life. So we continued for the remainder of that cold March weekend. I listened to discussions about labor stages with images that represented Black women, made rice socks with lavender (hot packs) with the trainees, and conversed about their motivations to become doulas. Attending this doula training was one of the last pre-COVID-19 experiences I had. Within a few days, the world and its outlook on the coronavirus had changed drastically. NYS had become an epicenter in the United States, and Central NY began to see cases and deaths related to the virus. Due to this situation, the doulas postponed the second weekend of training as they adapted the material virtually and rescheduled specific aspects, including planned guest speakers. Thus, this potential new cohort of doulas had to delay the completion of training and were unable to attend and support births in person.

During the beginning of COVID-19, the CBDs, like other birth workers in NYS, became worried about the state of maternal health, particularly for Black women. Some hospitals in the pandemic epicenter NYC would not allow pregnant women to bring labor support companions, forcing these women to labor alone (Davis-Floyd et al., 2020). Specifically, in mid-March 2020, New York-Presbyterian and the Mt. Sinai Hospital System briefly barred all visitors, including partners, in their labor and delivery unit after discovering that multiple pregnant and postpartum patients had COVID-19 (Hafner, 2020). Human rights and RJ advocates sounded the alarm, fearful that other hospitals would follow suit, and insisted that hospitals allow at least one support individual for birthing people. Due to the outrage, Governor Cuomo swiftly signed legislation to allow one support person for laboring women (van Syckle and Caron, 2020). However, there was a growing fear of hospital births because of possible virus exposure. Some CBDs pointed to a case in Syracuse’s neighboring city, Rochester, where a man was not honest about being symptomatic so that he would be allowed to visit his wife in the maternity ward. Shortly after giving birth, his wife also began showing symptoms, and it was then that the husband admitted he was symptomatic. This incident prompted preventative protocol: all visitors must now be temperature-checked twice daily, and everyone must wear face masks (Burke, 2020).

Soon, hospital birth began to seem higher risk than a home or birth center delivery because of its unknown and invisible dangers. This potential risk of hospital delivery was particularly problematic for the Black community, who, as previously noted, already suffered from higher maternal mortality and morbidity rates than their white counterparts. The virus exacerbated an already flawed maternal health care system for Black and Brown women. Since the start of COVID-19 in NYC, the CBDs pointed to several cases where Black women or infants suffered preventable deaths. One such woman was Amber Isaac, who tweeted concerns on April 17th about her maternity care after doctors did not communicate the outcome of her bloodwork for declining platelet count (Olumhense, 2020). On April 20th, Isaac learned she had HELLP syndrome, which complicates pregnancy, was induced a month early, and ultimately passed away on April 21st following her child's cesarean delivery (Olumhense, 2020). Another woman, Chrissy Sample, lost one of her infant twins at 24 weeks because she could not get an in-person appointment (Bobrow, 2020). Sha-Asia Washington died after being pressured to receive an epidural, which was not appropriately administered (Dickson, 2020). The CBDs discussed how these examples could quickly occur in Syracuse due to the restrictions on in-person support. One Black doula noted, "I think [COVID-19] is greatly impacting [Black maternal health] because it is limiting the amount of people that can be in the laboring room. And for me, more people is more witnesses." Consequently, the CBDs noted their concern for their Black clients and overall community due to the COVID-19 regulations in NYS.

Indeed, CBDs discussed their clients’ and other community members' interest in home birth or some other out-of-hospital (OOH) option. Nationally, the COVID-19 pandemic has motivated examinations into hospital birth’s safety compared to OOH or “community births” at home or in freestanding birth centers (Davis-Floyd et al., 2020). On a state level, there was interest in accommodating non-hospital providers to take on more clients (New York State (NYS), 2020). However, there are few OOH options in Central New York, as only a few midwives practice outside the hospital, and those were quickly flooded with calls for their services. Sequoia, who is also in nursing school, discussed the lack of midwives in the Syracuse area, stating, "When we're thinking about COVID-19 a lot of people are like ‘I want to have my babies at home...The issue is that we don't have a lot of homebirth midwives in Syracuse. We don't have *any* Black homebirth midwives in Syracuse.” The lack of Black and Brown midwives, and the small number of homebirth midwives in general in Syracuse makes community birth inaccessible to most Black and Brown people.

Other concerns stemmed from the fact that Black and Brown communities have faced more financial and medical losses due to COVID-19. Many point out that such communities have poorer health outcomes in general; Asteir, who is also a mother of three, summarized her take: “[COVID-19] certainly exacerbated the crisis of mortality and illness in Black and Brown communities. But I think for Black people specifically, COVID-19 feels like something else.” In other words, COVID-19 exacerbates pre-existing inequalities and health disparities in communities that already face tremendous systemic oppression, specifically within Black maternal health.

As a result of COVID-19, many doulas have turned to virtually supporting their clients to protect themselves. A CBD doula commented that "a bunch of Black doulas were like we’re not that essential. Like we're not essential enough to be risking our lives." In other words, the doulas understood they had to balance their own safety with the needs of their clients during COVID-19. As Black CBDs, they acknowledge the importance of their work with the Black community and the risk of COVID-19 to that same community's health. In the midst of the pandemic, the care and treatment of Black and Brown mothers remained centralized within these organizations. Through their doula care, the CBDs not only assessed what barriers they faced in delivering their care but also the barriers of their clients, fellow doulas, and greater community.

### Going Virtual

In response to hospital and state policies excluding them from the birthing room and to their own need for safety, many CBDs have begun to offer free virtual services, including prenatal, labor, and postpartum support. They have maintained activist engagement, have extended social media campaigns, and have continued dialogue online through “Live” on Facebook and Instagram to interact with followers. Another way in which the doulas reach their community and birthing clients is through free virtual childbirth courses.

Without initial approval or support from the state or hospitals to continue in-person doula care, virtual engagement became necessary. The CBDs began providing virtual doula support that ranges from being as simple as explaining specifics about infant health, or as significant as virtual labor coaching. VBI hosted a virtual training, offering guidance to many existing doulas who did not know how to provide virtual doula care. Through an Instagram post discussing the training, VBI stated:

We must ensure that the values of birth justice and human rights in childbirth are upheld and respected in the way pregnant people and their families are treated during this pandemic...Perinatal health disparities that impact the Black community do not disappear during a pandemic. They are further illuminated.

It is evident from this statement that the training would emphasize their overall mission to support Black and other marginalized pregnant women. The Zoom-based training event taught 15 doulas various fundamentals about offering doula care and support virtually. Every doula taught can mean dozens or ultimately hundreds of women served.

An additional essential effort I witnessed was the CBDs’ creation of virtual childbirth classes. They made a monthly series featuring four different topics for each class and CBD Sequoia delivered them via Zoom. The topics were the stages of labor, medicated and unmedicated comfort measures, breastfeeding, and postpartum care. During the first class, about 10 attendees joined with their partners, by themselves, with their families, in their living rooms, kitchens, bedrooms. By the end of the class, there were still topics to discuss, and many asked Sequoia to send the slides via email. Sequoia later reflected during our interview on the virtual childcare classes, stating, "I think it being online just eliminates… potential barriers, so more people are willing to attend." Indeed, each class that followed had a more extensive and larger presence, with about 20 individuals signing up by the final class—double the amount in the first one.

Another significant portion of the CBDs' virtual doula work focused on Black Lives Matter (BLM) and how this social movement connected to their work. This activist point of view was essential to include because it aligns with their Reproductive Justice (RJ) approach. I asked Sequoia to expand on that notion; she said, "There's been a lot of momentum around holding individual cops accountable and systems accountable. And I'm like we need this same type of energy around RJ and birth justice because we're losing too many Black moms, too many Latinx moms, too many people to preventable deaths… that's why we have to be thinking about how all these systems are connected." Consequently, CBDs attach great importance to advocacy and activism because these connect to Black peoples' overall endurance. Committing to this BLM movement was central to CBD work regardless of the pandemic, as this movement directly influences the Black lives of their community.

In facing the challenges generated by the pandemic, the CBDs virtually maintained this cornerstone activist component of their work. Following the police killings of George Floyd, Breonna Taylor, and Ahmaud Arbery, BLM protests of all forms sprang up throughout the nation and internationally. Both organizations posted multiple times and held campaigns in solidarity with this movement. In a social media post, D4Q captioned a photo of these three most recent victims of racism; the caption read, "When Black parents are scared to birth Black kids into an anti-Black society, one must understand how police violence is also a reproductive justice issue…we will proudly proclaim that Black Lives Matter!" In other words, the CBDs view their RJ approach to doula care as tied to the BLM cause of Black liberation and equity. Therefore, creating a virtual doula practice that makes space for BLM's discussion is significant to the CBDs and how they frame their work. This inclusion of BLM is significant as it demonstrates the connection the CBDs make between their RJ-centered doula care and the larger discussions happening in their community.

## The Future of In-Person Doula Care

After the initial virtual response to COVID-19, the CBDs were critical of how both NYS and specific hospital decisions directed the experience of birth and the CBDs' ability to care for their clients. The doulas shared apprehension that the policies to mitigate viral transmission would adversely affect maternal health experiences and outcomes for Black and other marginalized communities. I attended virtual meetings with CBDs, where there was discussion about a concern for "unnecessary inductions," "increased C-sections," and "rushed postpartum experience." Overall, the CBDs’ concern was that Black women face discrimination in healthcare settings even under normal circumstances, and this was exacerbated as the pandemic placed more stress on and gave more power to medical professionals. Sequoia described the frustration with current policies: "I've heard from quite a few Black women that the doctor's office only allows *them* to go in. So their doula can't go, their partner can't go. They have to wait in the car and FaceTime. So it's limiting the amount of witnesses and support that a person can have leading up to their delivery." In other words, there is a concern that Black women, who already face adverse maternal health outcomes, will not receive the proper support, which in turn could negatively impact their birth outcomes. For many Black women, this support is crucial to achieving positive maternal health outcomes.

In April 2020, Governor Cuomo assembled a task force to create maternal health care recommendations during COVID-19 (New York State (NYS), 2020). This task force recommended permitting doulas in addition to a personal support person into the birthing room. This official acknowledgment of doulas was a significant moment for all doulas in NYS, as the language of the recommendations described doulas as “essential.” While these state recommendations were certainly an improvement over the prior lack of consideration, the doulas still had apprehensions because, ultimately, the hospital had the authority to approve or decline a doula. The CBDs critically discussed the phrasing of the recommendation: "Exceptions should be made only in limited circumstances and based on clinical guidance, such as availability of [Personal Protective Equipment] PPE" (New York State (NYS), 2020). Many of the doulas view this as concerning because "decisions were in the hands of hospitals, not families." In other words, individual hospitals could have significant power in allowing or dis-allowing in-person doula care for specific patients, depending upon their ability to provide proper PPE.

Furthermore, many of the doulas criticized the contrast in the recommendations to admit doulas into maternity wards. On the one hand, the recommendations distinctly indicated that "doulas are considered an essential part of the support care team" (New York State (NYS), 2020). On the other, there was no recommendation about securing their entrance into hospitals or guaranteeing PPE. This lack of support was not unusual for the doulas, as they had long seen interest but no real commitment from local or state governments.

In discussing their specific concerns about the recommendation, the doulas were troubled by the barriers this lack of clarity may create. Asteir noted during our interview, "Why is it that in these three hospitals, doulas can't get in? Now they are saying that you need to show your certification if you show up for a birth. So I'm this person's support person and I have to validate myself with a certification that you really don't even honor." Sequoia agreed with this assessment, saying, "There is still a barrier because you have to quote 'prove' that you are a certified doula…it’s still that barrier of regulating who is a doula." In other words, the “proof” necessary to demonstrate that they are indeed certified doulas is a barrier for CBDs because there is an implication of regulation on who is an “official” doula. Thus, this is an obstacle for CBDs because they may need to validate their position as a doula to the hospital that may decide anyway to reject or discriminate against them.

Shortly after the statewide policy changed to allow doulas in the birth room, VBI and D4Q contacted a local labor and delivery nurse to host a question-and-answer session with the CBDs. The meeting was held on Zoom with 10 local doulas, allowed the nurse to provide insight into how doulas could affect their clients during labor, and gave the doulas the ability to ask questions, such as: How does the COVID-19 policy affect the birth experience? Have any doulas been present? What can a doula do to assist virtually? It was evident during this session that the doulas were concerned about whether or not the hospital would permit the doula’s presence and how they could ensure that they would be allowed to support their client. The nurse answered from her perspective, suggesting having the client inform her doctor and the maternity floor of her desire to have a doula present. The nurse emphasized the various factors that may influence the acceptance of doulas, such as the attending physician, the nurses on staff, and PPE availability. She noted that, to her knowledge, PPE was “limited but available” for doulas at her hospital.

Unfortunately, due to the limited amount of PPE at most hospitals, the doulas do not feel they can safely offer in-person care due to the close physical nature of continuous labor support. Asteir summarized the doulas' perspective, saying, "There is so much more that needs to get done if you are going to make statements like ‘doulas are essential’." Sequoia echoed this response, "Where is the conversation that's matching the risk?... We need no strings attached to funding but if we're not going to get it, PPE would be great." Many doulas can provide PPE for themselves, such as face shields, gloves, and cloth or disposable face masks. However, unless provided by the hospital, the doulas do not have access to medical-grade PPE, namely 95-masks, which significantly prevents the spread of COVID-19 during close contact. For Sequoia and the other doulas, the money that the state has proposed to support the doulas carries regulations from the state and does not entrust the money to organizations that have been working towards bettering the community. Indeed, the doulas commented that this is why community support and fundraisers are crucial for their work. Their words validate the lack of structural support in NYS that these CBDs are experiencing. Both women plainly stated their need to see real investment, whether monetarily, through ensuring PPE availability, or both, in committing to in-person doula care. Of course, COVID-19 is still a risk factor. Without vital structural support, many CBDs will not operate in-person.

Two other concerns for the NYS mandate are related to the partner's attendance and the doulas' personal lives. One doula discussed the stress some expectant mothers face if their partner cannot attend the labor and birth for the entire duration. NYS and other states do not allow support people to return once they have left. This can become a major stressor for both doulas and clients. One doula said, "If their partner has to work, I know that this was one issue with a lot of people. They were like I know my partner can be there, but they might not be able to be there three, four days that I'm gonna be in the hospital." Thus, there is still a fear that Black and other marginalized women may be giving birth and experiencing the postpartum period unsupported. For doulas, personal life concerns during COVID-19 include worries about their own children. Sequoia mentioned that "with COVID-19, it's like if you are a doula who had kids, you usually can just find a babysitter and go to your birth, but now it's like, is it safe to send your kids somewhere while you go support this mom?" Gaining in-person access to hospitals still leaves doulas facing the risk of infecting themselves or their loved ones. To be willing to accept this risk, the CBDs emphasize their need to have real investment from NYS showing that its officials believe in and promote doula work. Without this kind of concrete support from the state, CBDs do not feel that they can provide in-person doula care. Instead, their doula work, from individualized birth support to activist engagement, will continue online and will monitor the ability to practice in person.

For these doulas, virtual doula care in terms of continuous labor and delivery support is complicated. Most of the doulas have noted that they provide online prenatal and postpartum support with communication during delivery through assistance from their support partner via video-calling, texting, or phone calls. Sequoia noted that for doulas “supporting labor without [physically] being there is hard because that in-person part is key.” Instead, some of the doulas discussed their role during virtual labor support as guiding the support person present in the labor room. In this way, the doulas help coach the support person to fill in as a surrogate doula, give them advice on breathing methods, answer questions, or remind them of the massage techniques they had learned. Many of the doulas noted they used a smartphone to communicate with their clients during labor, but the clients did not always desire it. A doula with an upcoming birth told me that the expected “parents know to text or facetime me or whatever, when they want to talk or ask questions…but who wants to stay facetiming when you’re having a baby?” In this vein, it is evident that continuous virtual labor support may not be convenient or wanted by the expected parent, so they will only engage with the doula when they see fit. Thus, the doulas remain flexible depending on the needs or barriers of their community, clients, hospitals, and state guidelines.

## Conclusion

As a result of the COVID-19 pandemic, doulas have had to change their practices to keep themselves, their clients, and their communities safe. Thus, virtual doula practice has grown, with organizations like VBI, D4Q, and many more ensuring continual support. The interruption of in-person interaction did not diminish the D4Q or VBI doulas' motivation. Instead, they moved their work online and continue to provide virtual services, including monthly childbirth classes, while remaining vocal on activist issues that influence their communities and their clients. With some clients looking for out-of-hospital birth options, in the future doulas may be more able to operate outside of a hospital setting. This possibility may present new challenges but ultimately may have fewer barriers than the current hospital system under COVID-19 restrictions for CBDs. It remains to be seen how this system will change once the COVID-19 vaccine is widely available.

Despite the many barriers for CBDs in NYS, these two organizations navigated the obstacles to deliver RJ-based care that centers the need of the Black and Brown community in the city of Syracuse, specifically birthing mothers. As NYS cases of COVID-19 maintain a downward trend, CBDs may decide to re-initiate doula care in-person; yet the virus's course and the coming vaccine availability, along with local hospital and NYS restrictions, will ultimately weigh on the conclusive choice to re-start in-person doula work. Until then, VBI and D4Q will continue to reach their community and clients safely, virtually.

## Data Availability

The original contributions presented in the study are included in the article/Supplementary Material, further inquiries can be directed to the corresponding author.
